# Revealing the transcriptomic complexity of switchgrass by PacBio long-read sequencing

**DOI:** 10.1186/s13068-018-1167-z

**Published:** 2018-06-20

**Authors:** Chunman Zuo, Matthew Blow, Avinash Sreedasyam, Rita C. Kuo, Govindarajan Kunde Ramamoorthy, Ivone Torres-Jerez, Guifen Li, Mei Wang, David Dilworth, Kerrie Barry, Michael Udvardi, Jeremy Schmutz, Yuhong Tang, Ying Xu

**Affiliations:** 1 College of Computer Science and Technology, Jilin University, Changchun, China; 2 Department of Biochemistry and Molecular Biology and Institute of Bioinformatics, University of Georgia, Athens, GA USA; 3 BESC BioEnergy Research Center, Oak Ridge National Lab, Oak Ridge, TN USA; 4 Department of Energy Joint Genome Institute, Walnut Creek, CA USA; 5 HudsonAlpha Institute for Biotechnology, Huntsville, AL USA; 6 Noble Research Institute, LLC, Ardmore, OK USA

**Keywords:** Switchgrass, PacBio sequencing, Transcriptomic analysis, Alternative splicing, Plant cell wall

## Abstract

**Background:**

Switchgrass (*Panicum virgatum* L.) is an important bioenergy crop widely used for lignocellulosic research. While extensive transcriptomic analyses have been conducted on this species using short read-based sequencing techniques, very little has been reliably derived regarding alternatively spliced (AS) transcripts.

**Results:**

We present an analysis of transcriptomes of six switchgrass tissue types pooled together, sequenced using Pacific Biosciences (PacBio) single-molecular long-read technology. Our analysis identified 105,419 unique transcripts covering 43,570 known genes and 8795 previously unknown genes. 45,168 are novel transcripts of known genes. A total of 60,096 AS transcripts are identified, 45,628 being novel. We have also predicted 1549 transcripts of genes involved in cell wall construction and remodeling, 639 being novel transcripts of known cell wall genes. Most of the predicted transcripts are validated against Illumina-based short reads. Specifically, 96% of the splice junction sites in all the unique transcripts are validated by at least five Illumina reads. Comparisons between genes derived from our identified transcripts and the current genome annotation revealed that among the gene set predicted by both analyses, 16,640 have different exon–intron structures.

**Conclusions:**

Overall, substantial amount of new information is derived from the PacBio RNA data regarding both the transcriptome and the genome of switchgrass.

**Electronic supplementary material:**

The online version of this article (10.1186/s13068-018-1167-z) contains supplementary material, which is available to authorized users.

## Background

Switchgrass (*Panicum virgatum* L.) is a perennial grass native to North America and considered a major biofuel crop for cellulosic ethanol production, because of its strong adaptability and high biomass production [1–4]. Research on its net energy production and sustainability supports the economic feasibility in using the plant as a long-term biomass crop [5, 6]. As for any biofuel crop, overcoming biomass recalcitrance to deconstruction prior to conversion is the key challenge for this plant [7–9], to make its biofuel production economically feasible and competitive. In the past few decades, substantial efforts have been invested into genetic and genomic research of the plant [10, 11]. As of now, its genome has been sequenced although it is yet to be fully assembled into complete chromosomes. The most recent version of the genome (Pvir_v4) is 1165.7 Mb long, consisting of 139,331 sequentially ordered contigs [12]. Genes were annotated using both evidence-based approaches, i.e., cDNA, ESTs, and RNA-seq, and ab initio prediction [13], which have identified 91,838 distinct genes. 123,242 unique transcripts, including 31,404 AS transcripts, have been identified as of now [12].

Previous studies have suggested that ~ 60% of the multi-exon genes in plants harbor AS transcripts [14]. Compared to this number, substantially more AS transcripts are yet to be uncovered in the transcriptome of switchgrass. A key challenge lies in accurate reconstruction of full-length (FL) splicing transcripts from short sequencing reads [15].

The emergence of long-read sequencing techniques such as PacBio single-molecular technology promises more accurate elucidation of FL transcripts of all organisms, especially heterozygous polyploids like switchgrass [16]. Specifically, the PacBio technique eliminates the need for sequence assembly [17, 18] because of its ability to sequence reads up to 50 kbp long, hence providing direct evidence for splicing transcripts for the vast majority of plant genes. The technology has proven highly effective for unraveling the transcript diversity at complex loci [17], for accurate mapping of RNA sequences to the host genome [19] and for determining allele-specific expressions [20]. However, the technology has its own limitations: (a) it has high sequencing-error rates (~ 15%), predominantly *indel*s, compared to Illumina sequencers (~ 1%); and (b) it is of relatively low throughput, making it difficult to provide quantitative information about gene-expression levels at this point. Fortunately, the strengths and limitations of Illumina and PacBio techniques are highly complementary to each other. Together, they can potentially provide more accurate information about the transcriptome of a plant than either one alone [21].

Here, we present a transcriptomic analysis conducted using PacBio Iso-Seq technology [22], generated from six pooled tissue types: root, leaf blade, leaf sheath, internode, node, and flower. In parallel, Illumina paired short RNA reads, generated separately from ten un-pooled tissue types, were used as supporting data for our PacBio-based analyses. They are specifically used for (a) validation of splice junctions and AS events in PacBio transcripts; and (b) providing quantitative information for expression analysis.

Our analyses have generated the following information, which is also compared with the genes and transcripts annotated in switchgrass genome version 3 (Pvir_v3, a prefinished draft genome): (1) identification of 105,419 unique transcripts, covering 43,570 genes (42.7%) of Pvir_v3 (Note: we started our analysis when Pvir_v4 was not available) and 8795 non-Pvir_v3 genes, referred to as *previously unknown* genes, that are revealed by 9487 transcripts, 42.2% of which have homologous proteins in the NR database; (2) 60,096 AS transcripts of 16,642 genes; (3) 45,168 novel transcripts of 18,520 known genes; (4) 16,640 genes with exon–intron structures that differ to those predicted in Pvir_v3; 11,703 fusion transcripts [23] are predicted based on our PacBio data over the Pvir_v3 draft genome; (6) 1296 FL and numerous non-FL transcripts are not map-able to the switchgrass genome but together they are homologous to 7771 distinct proteins in one of the following organisms: sorghum, foxtail millet, and maize; (7) 96% of our predicted splice junctions are consistent with the Illumina data; and (8) 1549 distinct transcripts are predicted to be cell wall (CW) related, 639 of which are previously unknown. Overall, this is the first study of PacBio-based transcriptomic data of switchgrass, to the best of our knowledge.

## Results

### Mapping of FL transcripts to genomic DNA

We have identified 3042,460 reads of insert (ROI) (Table 1, Additional file 10: Figure S1) using the Iso-Seq Tofu pipeline (Additional file 10: Figure S2) from the PacBio RNA data. After removing 322,896 short reads (< 300 bp) and 17,119 artificial concatemers [24], 859,117 were identified as FL transcripts based on the presence of both 5′ and 3′ signals plus the polyA tails, and 1843,328 as non-FL transcripts. We noted that 47, 21, and 22% of the non-FL transcripts each miss all 5′, 3′ and polyA signals; 3′ and polyA signal; and 5′ signal, respectively (Additional file 11: Table S1). The following summarizes our analysis results of the FL transcripts.

**Table 1:** A summary of the initial and processed Iso-Seq data from six pooled tissue types

Library	# SMRT cell	#ROI	#FL	#Non-FL	#Reads < 300 bp	#Artificial concatemers	Mean size of FL (bp)	% FL	#Consensus sequences
Gel									
PB0938	2	57,613	17,273	33,863	6455	22	2019	30	5337
PB0939	2	60,270	10,318	41,604	8325	23	2911	17	2393
PB0940	2	35,226	485	2371	32,345	25	406	10	208
PB0941	2	60,887	17,463	11,806	31,566	52	524	29	5984
PB0942	2	103,211	31,359	48,990	22,817	45	1115	30	8779
SageELF									
PB0988	2	264,829	90,710	156,094	16,832	1193	2079	34	34,066
PB0989	2	313,987	91,557	205,554	15,603	1273	2640	29	30,392
PB0990	2	330,259	105,964	205,387	17,295	1613	2462	32	35,342
PB0991	2	266,928	89,911	154,177	21,440	1400	2012	34	29,121
PB0992	2	270,609	99,371	140,062	29,964	1212	1541	37	29,266
PB0993	2	290,900	109,275	138,236	41,563	1826	1118	38	30,534
PB0994	2	276,923	105,743	116,597	51,857	2726	823	38	27,245
PB0995	2	67,946	37,849	19,610	10,281	206	537	56	14,243
PB0996	2	273,102	15,683	249,333	6013	2073	6407	6	3169
PB0997	2	369,770	36,156	319,644	10,540	3430	4262	10	9694
Summary									
Total: 15	30	3042,460	859,117 (28.2%)	1843,328 (60.6%)	322,896 (10.6%)	17,119 (0.6%)	N/A	N/A	265,773

265,773 distinct FL consensus transcripts resulted from further processing using the Iso-Seq pipeline, which merges each group of highly similar sequences into one consensus sequence using ICE, followed by refinement of the consensus transcripts using Quiver in conjunction with 544,150 non-FL transcripts. These sequences were then mapped onto the Pvir_v3 genome [25]. The mapping results fall into three non-overlapping groups (as shown in Additional file 10: Figure S3):

G1: 245,758 transcripts (92.5%) each being uniquely mapped to one genomic locus;
G2: 18,719 transcripts (7.0%) each split mapped to two distinct genomic loci; and
G3: 1296 transcripts (0.5%) each showing no significant match to any genomic location, hence not mapped.

**Fig. 1:**
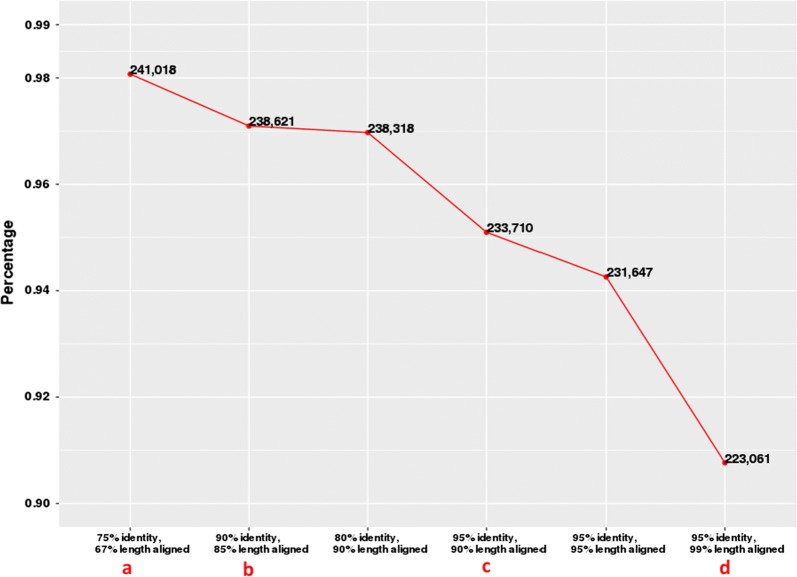
The percentage of the G1 transcripts satisfying each of the six thresholds defined along the *x*-axis. The four thresholds marked as a, b, c, d, are for high-quality alignment in human, maize, Amborella, and PacBio tutorial Iso-Seq analysis, respectively [18, 26, 27]

Figure 1 shows that the percentages of the G1 transcripts mapped to the Pvir_v3 genome using six different thresholds for the sequence-alignment similarity and sequence coverage, which were regarded as high-quality sequence alignments [18, 26, 27]. It is noteworthy that it may reflect either errors in the assembled genome or sequencing errors that some G1 transcripts do not have high-quality alignments with the genome. Further analyses are done only on those transcripts with high-quality alignments with the genome, specifically the 238,621 with at least 90% alignment identity and 85% sequence coverage.

We have also examined the 18,719 G2 transcripts, referred to as *fusion* transcripts [23] (see “Methods”), which give rise to 8850 distinct transcripts after removing redundant ones. Specifically, the 18,719 transcripts fall into 8850 groups of transcripts having approximately the same genomic coordinates by their alignment boundaries as shown in Additional file 10: Figure S4a; and only the longest transcript in each group is kept for further analyses. Of the 8850 transcripts, 6878 and 1972 are each split mapped to two inter-chromosomal and intra-chromosomal loci (Additional file 1), respectively. They cover a total of 6195 unique paired genomic loci, 2754 of which each have at least five Illumina pair-end reads linking the two genomic loci, hence indicating that these transcripts each represent one gene rather than fusion transcripts. In addition, we have also compared these fusion transcripts with available Sanger sequence data (see “Methods”), and found that 524 of them each match at least one Sanger-based transcript with its 5′ and 3′ ends linking the two genomic loci. Furthermore, 1360 of the 6195 pairs can be each mapped to one gene in the genomes of the three related species, having 90% alignment identity and 85% sequence coverage. In total, 55.4% of the 6195 genomic pairs each have at least one supporting evidence for being one gene rather than a true fusion gene, hence suggesting possible errors in the assembled genome. Detailed information about how the 8850 transcripts could be used to improve the Pvir_v3 genome assembly/annotation (shown in Additional file 10: Figure S4b) is given as follows:

1. Of the 4811 unique regions covered by the 6878 inter-chromosomal fusion transcripts, 1005 each link two chromatids of the same chromosome; 2090 each link two different chromosomes; 1639 each link a chromosome and a scaffold, and 77 each link two different scaffolds;
2. 1384 unique regions covered by the 1972 intra-chromosomal fusion transcripts link different regions in the same chromosome with the interval between the linked regions containing at least one gene in Pvir_v3, which is not part of the regions mapped by such transcripts; and
3. Of the 6878 and 1972 transcripts, 6068 and 1811 are homologous to 2526 and 805 distinct proteins, respectively, in at least one of three related genomes, sorghum, foxtail millet, and maize, determined by BLASTX 2.4.0 + [28] (*e* value < 1 ${\text{E}}^{ - 10}$) (Additional file 10: Figure S5a, b). Overall, 289 and 114 are chloroplast and mitochondrial genes, based on the NCBI definition [29], respectively.

The current understanding about this issue is while fusion genes have been found in microorganisms, they are believed to be rare in plants [30–33]. Based on this information and our comparison of the predicted transcripts with Illumina and Sanger-based transcript sequences as well as their homologs in other plants, we posit that most of the predicted fusion transcripts are probably not correct. Clearly, further validation work is needed to clarify this issue.

To assess if the G3 transcripts may encode proteins, BLASTX was used to compare these transcripts against protein sequences of the three species (*e* value < 1 ${\text{E}}^{ - 10}$). 369 of the 1296 transcripts (28.5%) were found to be homologous to 137 distinct proteins in at least one related genome (Additional file 10: Figure S6).

### Analyses of non-FL transcripts

We have also examined the 1843,328 non-FL transcripts identified by our pipeline (as shown in Additional file 10: Figure S3), aiming to derive additional information about the transcriptome as well as the genome of switchgrass. Note that the main difference between the FL and non-FL transcripts is if they each contain the 5′, 3′ signals and the polyA tails or not. The error rates in the non-FL transcripts are higher than those of the FL ones as no consensus-based error correction is applied to them [22].

We noted that the lengths of the non-FL transcripts are slightly shorter than those of the FL ones as shown in Additional file 10: Figure S7. Of the non-FL transcripts, 1274,642 (69%) can be mapped to the Pvir_v3 genome using GMAP, while 568,686 had no significant match to any genomic locations and, hence, were not mapped. Only the best alignment for each transcript is kept for further analyses, as described in the previous section. It is noteworthy that the medium base-pair mismatch rate across all such alignments range from 0.72 to 5.5% for the different sequencing libraries. We noted that the longer the library sequences, the higher the error rates, as expected (Additional file 10: Figure S8a, b). While the following analyses are focused on the 1274,642 transcripts, we noted that 10.9% of the 568,686 transcripts are homologous to 7743 distinct proteins in one of the three related genomes, determined using BLASTX and *e* value < 1 ${\text{E}}^{ - 10}$ as cutoff, 7634 of which are not homologous to any of the 137 proteins mapped by the G3 transcripts, which together gives rise to 7771 distinct proteins. Of these proteins, 411 and 157 are encoded by chloroplast and mitochondrial genes, respectively, and 1044 are uncharacterized proteins.

We have developed a statistical model for assessing if a transcript has a reliable mapping in the Pvir_v3 genome (see “Methods”), based on the known error rates of the sequencing libraries. Specifically, if the mismatch rate between a transcript and the mapped genomic segment is outside the range based on our model, we predict that this segment is not the DNA sequence of this transcript. Among the best alignments between the 1274,642 non-FL transcripts and their matched DNA, 176,328 (13.8%) are predicted not aligned with the correct DNA sequence, giving rise to 1,098,314 transcripts aligned with the correct DNA, based on our model. These transcripts were further corrected by their aligned genomic sequences using TAPIS and the default parameters [34]. At the end, 657,991 non-FL transcripts were kept after filtering out those alignments having inconsistent splice junctions between our prediction and Pvir_v3 annotation, determined using an SVM model trained based on SpliceGrapher [35]. Of these, 628,290 are each mapped to one genomic location and 29,701 are each split mapped to two loci as in G2. Following the same analyses on the G2 transcripts, the predicted fusion transcripts, representing 2853 unique ones, are mapped to 2549 genomic loci (Additional file 2) with 2191 being inter-chromosomal and 662 intra-chromosomal, respectively. Of the two sets, 1591 of 2549 are mapped to novel genomic locations compared to those mapped from the FL fusion transcripts. 979 of the 2549 (38.4%) each have at least five Illumina pair-end reads connecting the paired loci, strongly supporting our prediction.

### Analyses of the identified unique transcripts

Overall, 238,621 FL consensus transcripts and 628,044 non-FL transcripts are considered to have high-quality alignments with their matching DNA (with at least 90% sequence identity and 85% sequence coverage), giving rise to a total of 866,665 transcripts. Of these, 840,002 passed all criteria of the PASA pipeline for sequence assembly [36]. After further processing using the pipeline and removing 1908 short transcripts (< 100 bp, the minimum transcript length in Pvir_v3), 105,419 unique transcripts covering 60,616 unique genes resulted, 68,737 being FL and 36,682 non-FL ones, respectively (Table 2). Among them, 80,005 are intron-containing transcripts. To ensure that our prediction is of high quality, an independent program “collapse_isoforms_by_sam.py” [37] was used to validate our predictions. We found that 99.1% of the transcripts identified by this program were consistent with our prediction of unique transcripts.

**Table 2:** A summary of PacBio transcripts

Genome version	Type	#Transcripts	#AS transcripts	#Genes from PacBio transcripts	#Pvir_v3/4 genes covered by PacBio data	#Pvir_v3/4 genes not covered by PacBio data
Pvir_v3	FL	68,737 (43,487)	44,905 (31,773)	38,291 (1673)	34,111	
	Non-FL	36,682 (33,300)	15,191 (13,855)	29,856 (7262)	19,933	
	Total	105,419 (76,787)	60,096 (45,628)	60,616 (8795)	43,570	58,495
Pvir_v4	FL	68,742 (43,562)	44,932 (31,839)	38,258 (1825)	33,939	
	Non-FL	36,663 (33,318)	15,177 (13,846)	29,842 (7490)	19,699	
	Total	105,405 (76,880)	60,109 (45,685)	60,573 (9146)	43,201	48,637

The number inside each pair of parentheses represents the number of genes or transcripts not covered in Pvir_v3 or 4

Illumina short reads were mapped onto the Pvir_v3 genome using Tophat2 [38] and were compared with the genomic alignments of our 105,419 transcripts. 83.0 and 96.4% of the splice junctions in these transcripts were supported by at least five Illumina reads averaged over 66 Illumina samples and in at least one Illumina sample, respectively (Additional file 10: Figure S9a, b), hence offering a strong evidence for the high quality of these PacBio transcripts.

Overall, the 105,419 transcripts cover 43,570 Pvir_v3 genes, which were classified into nine distinct groups (Additional file 10: Figure S10), based on the type of the overlap between the aligned genomic regions and the exon–intron structure of the matched Pvir_v3 gene using Cuffcompare v2.2.1 [27, 39–41]:

1. 9487 transcripts (9.0%) are mapped to 8795 genomic loci not overlapping with any Pvir_v3 genes. To examine if these transcripts may indeed encode proteins, BLASTX was used to compare each with protein sequences in the NR database (*e* value < 1 ${\text{E}}^{ - 5}$), resulting in 4004 (42.2%) homologous proteins, of which 61, 20, 933, and 158 are chloroplast, mitochondrial genes, retrotransposons, and uncharacterized proteins, respectively (Additional file 11: Table S2);
2. 2500 transcripts (2.4%) each properly contain a Pvir_v3 gene;
3. 1463 transcripts (1.4%) are each located inside an intron of a Pvir_v3 gene;
4. The mapped genomic loci of 1228 transcripts (1.2%) each have one intron that contains a Pvir_v3 gene;
5. 28,632 transcripts (27.2%) each have the same exon–intron structures as the matching Pvir_v3 genes;
6. 6984 transcripts (6.6%) each overlap with part of but not the whole exon of a Pvir_v3 gene on the opposite genomic strand;
7. 2450 transcripts (2.3%) each overlap with part of but not the whole exon of a Pvir_v3 gene on the same strand;
8. 45,168 transcripts (42.8%) each share at least one splice junction with 18,520 Pvir_v3 genes, but differ at other splice sites; and
9. 5340 transcripts (5.1%) are each properly contained in the coding region of a Pvir_v3 gene.

In sum, (1–2) suggest the possibility that some genes might be missed or incorrectly predicted. (3–4) suggest the possibility that some regions in the current genome might be mis-assembled or some genes are missed or incorrectly predicted. (5) indicates that over one quarter of our predicted transcripts are consistent with the Pvir_v3 transcripts. (6–9) suggest that ~ 57% of our predicted transcripts are potentially novel splicing transcripts compared with Pvir_v3 transcripts.

**Fig. 2:**
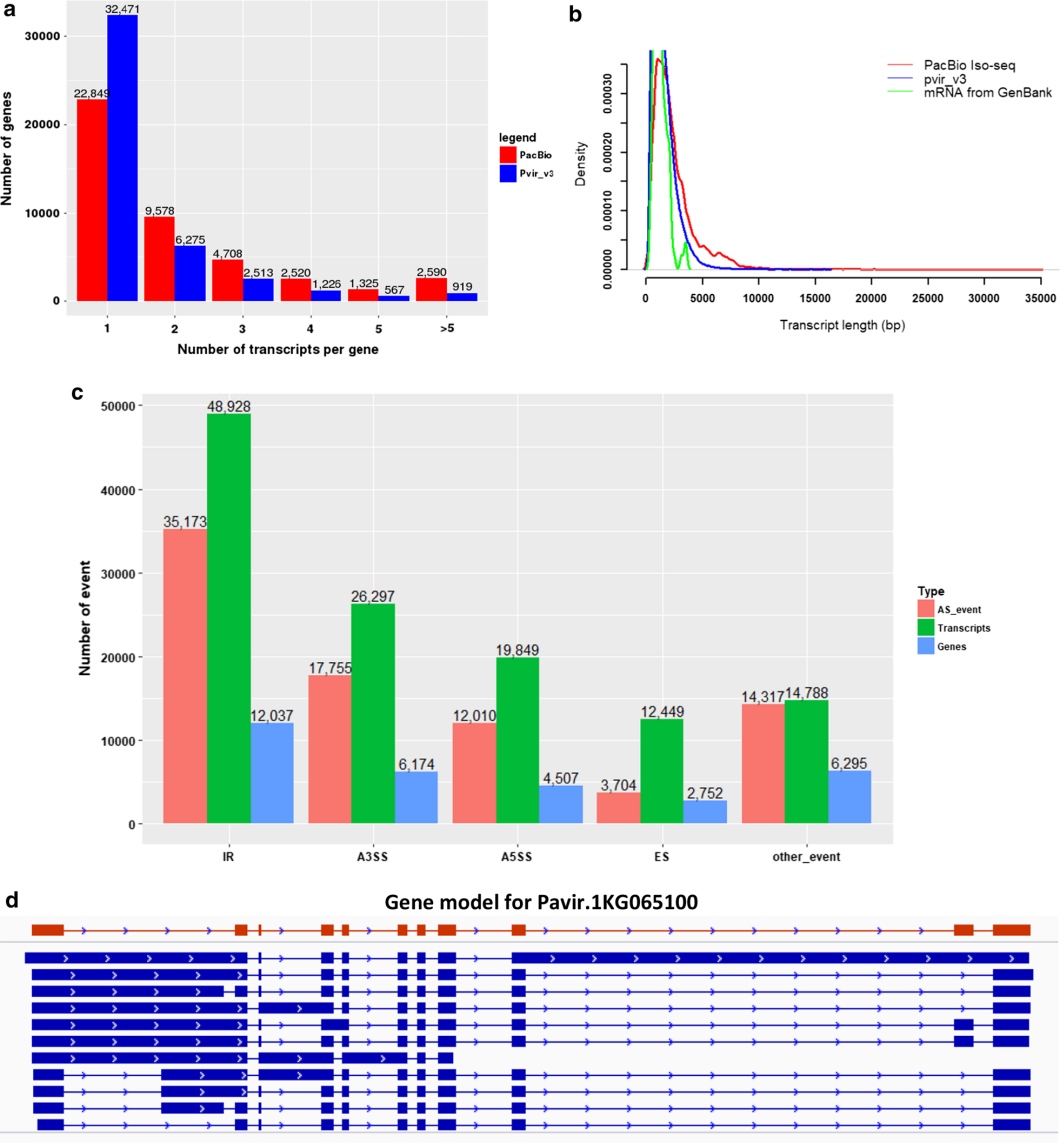
Characterization of the unique transcripts. **a** The number of transcripts per gene by PacBio data vs. those in Pvir_v3. **b** Length distribution of transcripts from PacBio sequencing, GenBank, and Pvir_v3, respectively. **c** The numbers of AS events, AS transcripts, and the relevant genes identified based on PacBio data. **d** An example of one gene with 11 AS transcripts. The transcripts in Pvir_v3 and PacBio are marked with red and blue color, respectively

Overall, our 76,787 transcripts each do not have exactly the same transcript in Pvir_v3. 59,942 of these transcripts are potentially novel AS transcripts (see “Identification of AS transcripts”) of known genes, and the remaining represent novel or mis-predicted genes. Furthermore, 21,072 of our identified transcripts are longer than the corresponding Pvir_v3 transcripts, predominantly at the two ends. As comparison, 104,731 Pvir_v3 transcripts are not identified by our analyses, and 22,423 Pvir_v3 transcripts are longer than our matching transcripts. We have also noted that (a) the number of distinct transcripts per gene derived by our analyses is higher than that by the Pvir_3 annotation (Fig. 2a); and (b) our identified transcripts tend to be longer than those in GenBank as well as by Pvir_v3 (Fig. 2b), specifically the mean length being 1,166 bp (switchgrass in GenBank), 1,569 bp (Pvir_v3), and 2,360 bp (PacBio), respectively.

At the gene level, 8795 of our predicted 60,616 distinct genes are not in the Pvir_v3 annotation, while 58,495 of the Pvir_v3 genes are not covered by the PacBio data (Table 2). The other 51,821 PacBio genes overlap with 43,570 Pvir_v3 genes. Of these, 16,640 have substantial overlaps by both predictions, and hence can be considered as the same genes. Some differences exist even among these predicted genes. Table 3 summarizes the main differences, including (i) different exon–intron structures; (ii) different genomic locations of the 5′ or/and 3′ boundaries; (iii) a PacBio transcript spanning more than one Pvir_v3 gene; and (vi) multiple non-overlapping PacBio transcripts covered by one Pvir_v3 gene.

**Table 3:** Difference in genes as annotated in Pvir_v3 and Pvir_v4 compared with PacBio transcripts, respectively

	#Genes in Pvir_v3	#Genes in Pvir_v4
Sequence span	348 (355)	340 (347)
Gene structures	6628 (8443)	6621 (8456)
UTR	10,447 (11,100)	10,365 (11,012)
Span multiple loci	291 (142)	288 (141)
Split	7 (14)	7 (14)
Total	16,640 (20,018)	16,544 (19,903)

“Sequence span” refers to genes with different starting or ending exon; “Gene structures” refers to genes with different exon–intron structures; “UTR” refers to genes with different UTRs; and “Span multiple loci” refers to multiple genes covered by one transcript. Other terms defined similarly. The number inside each pair of parentheses represents the number of PacBio transcripts used for making the comparison

### Identification of AS transcripts

We have applied the PASA pipeline [36] to infer AS events, namely intron retention (IR), alternative 3′ splice site (A3SS), alternative 5′ splice site (A5SS), exon skipping (ES), and other events (starts or ends in an intron, or alternative terminal exon) in the 105,419 transcripts. A total of 82,959 AS events are identified in 60,096 transcripts covering 16,642 genes (Fig. 2c). IR represents the predominant AS event (42%), A3SS is the second, and ES the least frequent, which are consistent with the published data on Arabidopsis and rice [14, 42].

Illumina reads, aligned to the switchgrass genome by Tophat2 [38] and processed by Miso [43], have been used to validate our predicted AS events. Overall, 85.7, 70.0, 60.0, and 85.8% of the predicted IR, A3SS, A5SS, and ES events have Illumina data support, respectively (Additional file 10: Figure S11). It is noteworthy that these numbers are not low, considering that the Illumina data used are collected from a different set of switchgrass samples. In addition, we have also used Sanger sequence data to validate the predicted AS events (see “Methods”). Overall, 2039 and 699 of the predicted IR and ES events have Sanger data support, 290 and 109 of which are different from Illumina validated, respectively.

Using the criterion that an AS transcript is a transcript containing at least one AS event, we predicted a total of 60,096 AS transcripts (Table 2), 76% being novel. Figure 2d shows one example of a gene annotated to produce a single transcript but found to have 11 AS transcripts.

### Quantification of PacBio transcripts

We have used the more quantitative Illumina data to estimate the expression levels of the 105,419 transcripts in each of the ten tissue types, from which the Illumina data are collected. The basic idea is to use the quantitative information of the Illumina reads that match each of the 105,419 transcripts to estimate the expression level of the transcript in each tissue type. Specifically, (i) for each transcript in each tissue type, the mapped Illumina reads were assembled by StringTie [44], using the exon–intron structure of the transcript as the reference, to estimate its FPKM; (ii) only those assembled transcripts sharing the same intron chains with those of the reference were kept; and (iii) only transcripts with FPKM $\ge$ 0.01 (a value chosen based on transcript coverage saturation analysis; Additional file 10: Figure S12a) in at least two replicates are regarded as successfully assembled.

At the end, 52,809 transcripts were assigned with expression levels across the ten tissue types with Illumina data. The first two principal components of these transcripts show that these transcripts can characterize the specificity and similarity between different tissue types (Additional file 10: Figure S12b). We have examined 3190 tissue-specific transcripts, 2601 which are novel. Table 4 summarizes the number of PacBio transcripts expressed in each of the ten tissue types.

**Table 4:** A summary of transcripts predicted to be expressed in each tissue type

Tissue type	#Expressed genes	#Transcripts	#AS transcript	# Tissue-specific transcripts	#CW-related transcripts	#lncRNAs	#TFs
Crown	31,256 (1177)	41,772 (17,769)	23,620 (11,506)	128 (106)	916 (155)	1156 (1053)	1813 (617)
Leaf blade	29,081 (1219)	39,747 (17,626)	23,306 (11,654)	248 (213)	651 (115)	1190 (1090)	1642 (592)
Leaf sheath	30,517 (1222)	41,689 (18,418)	24,251 (12,198)	215 (181)	787 (143)	1191 (1086)	1731 (616)
Node	31,294 (1243)	42,329 (18,421)	24,262 (12,070)	170 (138)	889 (153)	1197 (1090)	1798 (626)
VB	28,003 (1006)	36,926 (15,261)	21,247 (9934)	114 (94)	721 (120)	949 (858)	1526 (523)
Inflorescence	31,365 (1258)	42,298 (18,468)	23,966 (11,900)	219 (188)	890 (143)	1260 (1151)	1817 (614)
Root	32,133 (1255)	43,787 (19,288)	24,951 (15,563)	407 (294)	966 (180)	1227 (1126)	1867 (663)
Seed DAP	33,174 (1458)	45,985 (21,229)	26,328 (13,768)	1007 (839)	885 (163)	1460 (1335)	1925 (679)
Seed germ	30,905 (1212)	42,015 (18,519)	24,101 (12,100)	317 (260)	867 (162)	1199 (1094)	1708 (593)
Shoot	33,067 (1350)	45,553 (20,350)	26,095 (13,330)	365 (288)	979 (167)	1342 (1224)	1962 (691)
Shared by all tissue types	22,948 (729)	28,623 (10,666)	16,245 (6743)	N/A	490 (67)	686 (614)	1088 (351)

The number inside each pair of parentheses represents the number of genes or transcripts not covered in Pvir_v3

### Identification of CW-related transcripts

Tblastn was used to homology-map known CW-related proteins involved in cell wall construction and remodeling in Arabidopsis, maize, and rice to the 105,419 transcripts, respectively, as stored in the Cell Wall Genomics database [45] (*e* value $\le 1{\text{E}}^{ - 20}$ and b-score $\ge 93{\text{\% }}$ as cutoffs) [46] (Additional file 10: Figure S13). 1549 distinct PacBio transcripts of 1077 genes are homologous to CW-related transcripts (Additional file 3), of which 639 are novel transcripts of known genes of Pvir_v3 and 464 are novel AS transcripts. 553 of the 1077 genes (51%) significantly enrich the lignin, xyloglucan, and hemicellulose metabolic processes (*p value *< 0.01) (Additional file 10: Figure S14a, b, c). Table 5 summarizes the five CW synthesis-related functional categories that the 1549 transcripts fall into, with Table 4 detailing the CW-related transcripts expressed in each tissue type.

**Table 5:** Functional categories into which the 1549 predicted CW-related transcripts fall

Functionality	#Arabidopsis homologs	#Maize homologs	#Rice homologs	Total
Substrate generation	184 (10)	220 (14)	190 (14)	252 (19)
Polysaccharide syntheses and glycosyl transferase	253 (16)	411 (37)	336 (27)	442 (45)
Secretion and targeting pathways	81 (1)	N/A	N/A	81 (1)
Assembly, architecture and growth	285 (31)	357 (43)	322 (38)	436 (50)
Differentiation and secondary wall formation	94 (6)	158 (14)	26 (3)	166 (16)
Signaling and response mechanisms	115 (23)	135 (19)	20 (3)	185 (35)
Overall	1008 (87)	1272 (126)	889 (85)	1549 (165)

The number inside each pair of parentheses represents the number of non-FL transcripts. Note: N/A indicates there is no matching proteins in the database

We anticipate that these novel CW-related transcripts will provide useful candidates for studying plant cell wall synthesis and remodeling processes.

### Prediction of lncRNA

We have predicted which of the 105,419 transcripts may encode lncRNAs using the following procedure: (a) select from the 105,419 transcripts 13,021 candidates of at most 350 bp long each contained inside one open reading frame in the genome [47]; (b) remove from the candidate list all 6602 transcripts that encode proteins, as determined using BLASTX (*e* value < 1 ${\text{E}}^{ - 10}$) against the protein sequences in the three related species (Additional file 10: Figure S15a); and (c) select those having high non-coding RNA scores given by coding potential calculator (CPC) [48], resulting in 5165 strong and 1119 weak candidates for lncRNA. Further analysis is conducted on the 5165 strong candidates.

We noted that (i) 40% of the predicted lncRNAs were in intergenic regions, 9% in introns, 14% on the antisense strand, and 37% on the sense strand of protein-encoding regions [note: these are not homologous to those in the above (b)]; (ii) 2% of the predicted lncRNAs are homologous to known lncRNA in the three related genomes (Additional file 10: Figure S15b), which is not surprising knowing that RNA genes tend to not have sequence-level conservation; (iii) 71% are single-exon genes (Additional file 10: Figure S15c); and (iv) their FPKM values were lower than those of protein-coding genes (Additional file 10: Figure S15d). All are consistent with published studies [49–53]. Additional file 4 gives the list of the coordinates of the predicted lncRNAs in Pvir_v3. The expression levels of these IncRNAs in each tissue type are summarized in Table 4.

### Prediction of transcription factor (TF)

3205 transcripts are predicted to be TFs by scanning the HMMs of all the 56 families of TFs given in PlantTFDB 4.0 [54] against the 105,419 transcripts, which fall into 54 TF families (Additional file 10: Figure S16 and Additional file 5). To validate our prediction, 489 switchgrass TFs were collected from the literature [55–57], 331 of which are in our prediction, providing strong evidence for our prediction. The TFs expressed in each tissue type are summarized in Table 4.

## Discussion

### PacBio long reads vs. Illumina high-throughput short reads

From our analyses and comparisons, we see strengths as well as limitations of the PacBio data. Reliable identification of FL transcripts is clearly an advantage of the PacBio data, which has enabled us to infer considerably more AS transcripts than before. On the other hand, the relative lack of quantitative information in PacBio data vs. Illumina data is a weakness of the technology. Here, we have made some effort to integrate the information derivable from both the PacBio and the Illumina data as the two data types available to us are not collected from the same tissue samples. This represents an area where bioinformatics techniques can play a major role in optimally integrating information from the two data types, to offer both reliable and quantitative information for transcriptomic analyses of plants.

### Identification of alternative splicing in switchgrass

Our analysis detected 213,678 splice junctions in 105,419 transcripts, 32,508 of which (15%) were novel compared to the Pvir_v3 annotation. Over 96% of these junctions are supported by Illumina data, providing high confidence of our identification. We have estimated the percentage of the intron-containing genes that may encode AS transcripts by calculating the ratio between the number of such genes with at least one AS transcript and the number of such genes in our PacBio data, which is 47.4% having AS transcripts. In addition, 57.4% of the PacBio genes with at least two introns encode AS transcripts. Although this number is consistent with data provided in published studies [14, 58], we suspect that it is still an underestimate of the actual percentage as we used only six tissue types at the R1 stage under normal growth conditions. We expect that more AS transcripts-harboring genes may be revealed when more tissue types across more developmental stages under stressful conditions are subject to transcriptomic analyses.

### Pvir_v3 *vs.* Pvir_v4 of the switchgrass genome

JGI has recently released a new version, Pvir_v4, of the assembled genome along with its new annotation [12]. Since a vast amount of the computing and analysis work presented here was conducted before the public release of Pvir_v4, our presentation was focused on Pvir_v3.

We have run PASA [36] on the 866,665 PacBio transcripts (238,621 FL and 628,044 non-FL) against Pvir_v4 using the same parameters as against Pvir_v3, and got 105,405 unique transcripts (Table 2). The following differences between the two versions are observed:

1. 104,238 of the 105,419 transcripts (98.9%) match the same genomic sequences in Pvir_v4 vs. Pvir_v3, except for changes in their sequence coordinates. Among the remaining 1181 transcripts, the majority (878) have some minor differences in their sequence alignments and 296 are largely the results of regions that were N-ed out between the two versions (Additional file 10: Figure S17). Additional file 6 provides a mapping of the genomic coordinates between genes in the two versions, Additional file 7 gives the mapping between the corresponding transcripts in Pvir_3 and v4, and Additional files 8 and 9 provide the coordinates of 105,419 and 105,405 assembled transcripts against Pvir_v3 and Pvir_v4, respectively; and
2. We have also examined the 18,719 FL and 29,701 non-FL transcripts that are each split mapped to two distinct loci in Pvir_v3 and how they are mapped to Pvir_v4. We noted that 8803 such FL transcripts remain as fusion transcripts covering 6123 unique paired genomic loc, 41% of which have Illumina reads support, with 1962 being intra-chromosomal and 6841 inter-chromosomal, while 47 FL transcripts are each now mapped to one genomic locus or unmapped due to genomic rearrangements or regions that were N-ed out. Similarly, 2834 non-FL transcripts remain as fusion transcripts covering 2531 unique genomic loci, 43% of which are validated by Illumina reads, 643 and 2191 being intra-chromosomal and inter-chromosomal, respectively, while 19 non-FL transcripts are each now mapped to one genomic locus or unmapped due to genomic rearrangements or regions that were N-ed out. Furthermore, 10,083 Pvir_v3 genes are removed in Pvir_v4 due to their low prediction confidence scores and 144 are removed due to sequence changes in the reassembled genome of Pvir_v4.

## Conclusion

Through integrative analyses of PacBio- and Illumina (and limited Sanger)-based transcriptomic data, we were able to reliably infer splicing isoforms and their expression levels at a genome scale, by taking advantage of the strong complementary nature of the two data types. In addition, such analyses also provide highly useful information for guiding further improvement in the partially assembled genome of switchgrass.

## Methods

### Plant samples

Vegetative clones of switchgrass genotype Alamo AP13 obtained by splitting tillers were grown in 3-gallon pots with Metro-Mix 830 soil in a greenhouse with a 16-h light photoperiod (6:00 am–10:00 pm) with supplementary lighting from parabolic aluminized reflector lamps (average 390 μE/m2/S1) and relative humidity 77–22% (average 51%). The temperature in the greenhouse ranged from 25 to 29 °C (average 26 °C). Plants were watered three times per week, and fertilizer (Peter’s Fert 20-10-20, 100 ppm) was applied during the last watering each week. A whole tiller consisting of leaves, leaf sheaths, internodes, nodes, and flowers and roots were collected at the R1 developmental stage [59] and used to do PacBio sequencing. Ten tissue types—seed across three germination stages, root, shoot, leaf shade, leaf sheath, nodes, vascular bundle, crown, inflorescence, seed and flower across seven developmental stages of seed according to the same criteria described in [59]—were used to do Illumina sequencing (Additional file 11: Table S3).

### RNA preparation for PacBio sequencing

A whole tiller, including leaves, leaf sheaths, internodes, nodes, and flowers, was collected at the R1 development stage [59] and frozen immediately after resection in liquid nitrogen. Roots were collected and frozen in liquid nitrogen separately. Total RNA of the above-ground tissues was isolated using RNeasy Plant Mini Kit [60]. Total RNA of roots was isolated from roots using TRI REAGENT [61]. To eliminate residual genomic DNA contamination, RNA samples were treated using Turbo DNase and then cleaned up using RNeasy MinElute Cleanup Kit [60]. The cleaned RNA of the above-ground tissues and roots was mixed together.

### PacBio library construction

The first-strand cDNA was synthesized using SuperScript II (Invitrogen). The 3′ dT primer (5′-TAGTCGAACTGAGATCTCCAGCAGT_30_VN -3′) and total RNA were first incubated at 72 °C for 3 min and then the 5′ primer (5′-TAGTCGAACTGAGATCTCCAGCAGTACrGrGrG-3′) and SuperScript II were added into the reaction mix for reverse transcription and template switching. The reaction was performed at 42 °C for 90 min, followed by 50 °C for 2 min and 42 °C for 2 min for 10 cycles on a thermocycler. After the first-strand reaction, the cDNA (Additional file 10: Figure S18a) was amplified using a PCR primer (5′-TAGTCGAACTGAGATCTCCAGCAG-3′) and KAPA HiFi HotSart ReadyMix (KAPA).

Two size-selection procedures were used: (1) a set of libraries was made from the cDNA generated from five cycles of PCR and gel selection, and different size fractions (cDNA < 0.5 kb, 0.5–1 kb, 1–2 kb, 2–3 kb, and 3–6 kb) were collected from a 0.8% agarose gel and purified using the Zymoclean Large Fragment DNA Recovery Kit (Zymo) (Additional file 10: Figure S18b); and (2) to reduce bias towards short reads, a second procedure of size selection was performed on a Sage Science Electrophoretic Lateral Fractionator (ELF), resulting in the isolation of 10 discrete size fractions. cDNA from 10 cycles of PCR was loaded onto a 0.75% cassette (Sage), and size-based separation mode was used to select cDNA from 500 bp. cDNA fractions > 5 Kbp were collected for additional 10 PCR cycles and ELF selection to enrich long reads (Additional file 10: Figure S18c).

After size selection, the collected cDNA fractions were treated with DNA damage repair mix [62], followed by end repair and ligation of SMRT adapters using the PacBio SMRTbell Template Prep Kit to create PacBio libraries. These two size-fractional library sets selected by gel and SageELF were sequenced on the PacBio RSII platform using P5-C3 and P6-C4 chemistry with 4 h movies, respectively.

### Illumina RNA-Seq library construction

Plate-based RNA sample prep was performed on the PerkinElmer Sciclone NGS robotic liquid handling system using Illumina’s TruSeq Stranded mRNA HT sample prep kit utilizing polyA selection of the mRNA, following the protocol outlined in Illumina’s user guide [63]. This is done with the following conditions: the total RNA starting material was 1 μg per sample and 10 cycles of PCR was used for library amplification. The prepared libraries were then quantified using KAPA Biosystem’s next-generation sequencing library qPCR kit and run on a Roche LightCycler 480 real-time PCR instrument. The quantified libraries were then multiplexed into pools of six libraries each, and the pool was then prepared for sequencing on the Illumina HiSeq sequencing platform utilizing a TruSeq paired-end cluster kit (v4) and Illumina’s cBot instrument to generate a clustered flow cell for sequencing. Sequencing of the flow cell was performed on the Illumina HiSeq 2500 sequencer using TruSeq SBS sequencing kits, v4, following a 2 × 150 indexed run recipe. These 66 libraries are of high quality since the biological replicates have strong correlations among themselves, achieving Spearman’s correlation > 0.9 (Additional file 11: Table S4).

Illumina data were used to validate and quantify the PacBio-based transcripts. Although the Illumina data were collected from samples different from those from which the PacBio data were collected, we argue that such validation is valid for the following reasons: (1) the Illumina samples cover the types of switchgrass tissues for the PacBio data collection; and (2) the types of proteins, hence their mRNA types, including AS transcripts, in the two matching tissue types, possibly at different developmental stages and detailed nutrient conditions, should be largely the same.

### Data collected from the public databases

The switchgrass genomic sequences Pvir_v3.1 and v4 [12] were downloaded from JGI. Protein sequences, transcript sequences, and the genomic sequences of the three related species—sorghum (v3.1.1), foxtail millet (v2.2), and maize (5b+)—were also downloaded from JGI [64–66]. In addition, 130 published FL transcripts of switchgrass were collected from GenBank [67]. 35,660, 28,588, and 104,831 high-quality Sanger sequences of switchgrass genotype Alamo AP13 were collected from the literature [68] and used in our validation analyses. The sequences of 489 TFs of switchgrass were collected from the literature [55–57]. The lncRNA sequences of three related species were downloaded from the GREENC database [69, 70] and the PNRD database [71, 72], respectively. The Gene Ontology (GO) annotation for the three related species (sorghum v3.1.1, foxtail millet v2.2, maize 5b+) were downloaded from PlantTFDB 4.0 [54, 73], since which has latest gene annotation. The sequences of 922, 987 and 705 CW-related proteins of Arabidopsis, rice, and maize were downloaded from Purdue Cell-Wall-Genomics Database [45, 74], respectively.

### A computational pipeline for identification of consensus FL transcripts

The sequenced PacBio ROIs were selected using ToFu (version 2.3.0) [22] with the following parameters: minimum full pass > 0, minimum length > 300 and prediction accuracy > 75%. Then, the ROIs were classified into circular consensus sequences (CCS) and non-CCS subreads by ToFu based on the presence of sequencing adapters or not. The CCS subreads were deemed to be FL transcripts if they each have both the primer sequences (the 5′ and 3′ sequences) and the polyA tail signal. It is noteworthy that the mean number of sequencing passes of the FL transcripts for each PacBio library ranges from 5 to 25 (Additional file 10: Figure S19). Then, a clustering algorithm, ICE, was applied to the all FL transcripts to get consensus transcripts, which groups them into clusters based on the sequence similarity and generate a consensus sequence for each cluster. Then Quiver was used to polish the consensus transcripts to give rise to the high-quality FL transcripts with $\ge$ 99% post-correction accuracy.

### A statistical model for estimation of the mismatch error rate

We have designed a statistical model to check if a given transcript is correctly mapped to its DNA sequence in the genome, based on the known mismatch rate in the raw PacBio sequences. We have the following assumptions in the model: (i) mismatch errors are independent of each other [75]; and (ii) the probability *p* in having a sequencing error in any position is a fixed value between 0.0072 and 0.055 in our study, based on the error-rate distribution observed for size-fractional libraries (Additional file 10: Figure S8a). The following model is used to calculate the probability (*p* value) that the number of errors in a sequence of *L* bps is at least *K*:

$$ 1 - \varPhi \left( {\frac{K - Lp}{Lp(1 - p)}} \right), $$

where $\varPhi(t)$ is the standard Gaussian distribution and *K* is a user-specified positive value. Based on our calculation, the probability (*p* value) that *K* is larger than $L(p + 0.03)$ was small enough to be regarded as mis-alignment (Additional file 10: Figure S20).

We have assessed the performance of the model using Sanger-based RNA sequences collected on switchgrass tissues (see Data) as follows. We first mapped the Sanger-based RNA sequences to the genome of Pvir_v3 using GMAP (version 2015-1-20) [25] (parameters: -f samse -t 30 -B 5 –sam-use-0M), which are known to have 99.999% per-base accuracy [76]. Hence, such mapping results are considered as correct mapping (Additional file 10: Figure S21). Then for each of the nine chromosomes, we mapped the Sanger sequences of the other eight chromosomes to this chromosome by our model, which gives rise to *false positive* rate at 17.3% across all nine chromosomes (Additional file 11: Table S5). Similarly, we mapped the Sanger sequences to their correct chromosomes, and we found that the *false negative* rate at 4.74%.

### A computational pipeline for processing non-FL transcripts

The best alignment for each non-FL transcript against genomic DNA of Pvir_v3, determined by GMAP (2015-1-20) [25] with the following parameters: -f samse -t 30 -B 5 –sam-use-0M, was kept for further analyses. A transcript is filtered out if it was mapped to a homologous region rather than its correct genomic location, assessed using our error-rate estimation model. A process is then applied to remove low-quantity PacBio transcripts, including those each consisting of ‘N’s, ambiguous junction sites, or > 10% of sequencing errors (indel or mismatch). Then, the TAPIS (version 1.2.1) pipeline [77] with the default parameters is applied to the remaining transcripts for error correction based on the aligned genomic sequence. Furthermore, a SVM-based model is trained and used to filter out transcripts containing false splice junctions in the Pvir_v3 genome using “build_classifiers.py” in SpliceGrapher (version 0.25) [35] with the following parameters: -d gt, gc -n 5000. It is noteworthy that the ROC scores for splice site consensus—AG, GC, and GT—are 0.94, 0.93, and 0.95, respectively, based on our prediction assessment of splice junctions in Pvir_v3, where 1.0 is the highest possible score for such a prediction.

### De novo identification of unique transcripts and annotation comparisons using PASA

The high-quality transcripts, FL or non-FL, are fed into the PASA (version 2.0.2) pipeline [36] with the following parameters: MIN_PERCENT_ALIGNED = 85, MIN_AVG_PER_ID = 90, and –ALIGNERS gmap, for identification of unique transcripts. Firstly, these transcripts are aligned to the genomic DNA of Pvir_v3 (4) by GMAP [25], and then each valid alignment satisfying the following criteria: at least 90% sequence identity and 85% transcript length aligned; consensus splice sites at all inferred intron boundaries are used for the assembly of spliced alignments. Overall, 73% of the PASA assemblies are each the result of collapsing at least two transcripts (Additional file 10: Figure S22), indicating that each splice junction was not detected by accident. Each assembly that contains at least one FL transcript is termed FL-assembly, otherwise, non-FL-assembly.

The annotation comparison module in PASA was applied to conduct transcript-level comparison. Gene models from Pvir_v3 (4) are loaded as the original annotation on the first cycle of annotation comparison. PASA-assembled transcripts were then used as input transcripts. Three cycles (locus difference coverage saturation analysis at Additional file 11: Table S6) of transcript loading, annotation comparison were conducted to maximize the integration of the information about transcript alignments into the prediction of gene structures.

### Illumina data analysis

Tophat (version 2.1.1) [38] was used, with default parameters, to map Illumina reads from ten tissue types onto the Pvir_v3 genome. The mapped short reads were assembled into transcripts using StringTie (version 1.0.4) [44] with the default parameters and the identified PacBio transcript as the template. The FPKM values were used to quantify each assembled transcript. A transcript was regarded as successfully assembled if its exon structure was the same as matching PacBio transcript and FPKM value $\ge$ 0.01.

### Identification of AS events

Illumina reads were used as validation for each predicted AS event using Miso (version 0.5.4) [43] with the following options: –overhang-len 8 –read-len 150, which provides information about which Illumina reads are included and which are excluded to be consistent with each predicted AS, referred to as the *inclusio*n and *exclusion* reads for the AS event. The following criteria are used to determine if an AS event is supported by the Illumina reads: #inclusion reads $\ge$ 1, #exclusion reads $\ge$ 1, and #inclusion reads + exclusion reads $\ge$ 10.

In addition, Sanger-based RNA reads, mapped by GMAP [25] against Pvir_v3, were also used to validate the predicted AS events. Each IR event whose intron region covered by one exon of a Sanger transcript or each ES event whose exon region covered by one intron of a Sanger transcript is regarded as validated by Sanger data.

### Prediction of fusion transcripts

A transcript is considered a *fusion* transcript if the following criteria were met: (1) the transcript is mapped to two or more distinct protein-encoding loci in the genome; (2) each such locus aligns with at least 5% of the relevant transcripts; (3) the combined alignment coverage across all the matched loci should be at least 85% of the transcript; and (4) two mapped loci are at least 100 kbp apart (value used to detect fusion transcripts in maize PacBio data analysis [27]).

Predicted fusion transcripts are validated against Illumina short reads. Specifically, a fusion transcript is considered as validated if for each pair of predicted fusion regions there are paired Illumina reads that are mapped to the corresponding two regions, determined by Tophat2 [38].

### Functional prediction of PacBio transcripts

We have predicted the function of each selected PacBio transcript as follows: (i) blastn was used to bi-directionally map the transcript to the transcript sequence of the three related species; and (ii) functional prediction of each mapped PacBio transcript using the function of its mapped genes; specifically, only bi-directional best hits with *e* value < 1 ${\text{E}}^{ - 10}$ and b-score > 90% were considered as having the same GO function. The R package topGO [78] was used to do GO-based function prediction.

### Identification of lncRNA from PacBio transcripts

Transcripts with CPC score < -1 (predicted by CPC [79], regarded as strong non-coding RNA) were predicted as lncRNAs, where CPC score for these lncRNA candidates range from − 1.5515 to − 1.00007. To check if a predicted lncRNA is species specific, we have BLASTed it against the annotated lncRNAs in the reference species (*e* value $\le$ 1 ${\text{E}}^{ - 10}$).

## Additional files

**Additional file 1.** Genomic locations mapped by 8850 FL fusion transcripts.

**Additional file 2.** Genomic locations mapped by 2853 non-FL fusion transcripts.

**Additional file 3.** CW-related transcript list and functional prediction.

**Additional file 4.** Genomic coordinates of the predicted lncRNA transcripts.

**Additional file 5.** The TF list and matching families.

**Additional file 6.** Mapping of the genomic coordinates between the same genes in Pvir_3 and v4.

**Additional file 7.** Mapping between the corresponding transcripts in Pvir_3 and v4.

**Additional file 8.** The coordinates of 105,419 assembled transcripts in Pvir_v3.

**Additional file 9.** The coordinates of 105,405 assembled transcripts in Pvir_v4.

**Additional file 10: Figure S1.** The distribution of the sequence lengths of ROI for each size-fractional library. **Figure S2.** The workflow of the Iso-seq Tofu pipeline. **Figure S3.** The workflow of the transcriptome analysis and assembly pipeline for PacBio transcripts. **Figure S4.** An illustrative example of fusion transcripts. **Figure S5.** A Venn diagram of our predicted 8850 fusion transcripts showing homology to proteins of the three related species. **Figure S6.** A Venn diagram of 1296 HQ FL transcripts showing homology to proteins of the three related species. **Figure S7.** Length distribution of FL and non-FL transcripts in each library. **Figure S8.** The base-pair mismatch rate of non-FL PacBio sequences in each size-fractional library. **Figure S9.** Validation of PacBio transcripts by Illumina-based RNA-seq reads. **Figure S10.** Illustrative examples of nine groups of PacBio transcripts. **Figure S11.** Validation of AS events by Illumina reads. **Figure S12.** Quantification of PacBio transcripts. **Figure S13.** Thresholds for selection of two parameters: the *e* value and the proportion of best-hit bit-score. **Figure S14.** GO-based pathway enrichment analysis of our predicted CW transcripts. **Figure S15.** Characterization of our predicted lncRNAs. **Figure S16.** 54 TF families identified from FL and non-FL transcripts. **Figure S17.** The distribution of the ratio between sequence lengths of 1174 transcripts in Pvir_v3 vs. Pvir_v4. **Figure S18.** Size-dependent cDNA libraries of Switchgrass. **Figure S19.** Number of sequencing pass for FL transcripts in each library. **Figure S20.** The probability (*p* value) that the number of errors (*K*) in a sequence of length *L* is larger than ($L(p + 3{\text{\% }})$). **Figure S21.** The distribution of base-pair mismatch rate of Sanger sequences. **Figure S22.** The bar plot of the number of transcripts collapsed by each PASA assembly.

**Additional file 11: Table S1.** A summary of six classifications based on the presence of 5′ primer, 3′ primer and polyA signal in each non-FL transcripts of different size-fractional library sets. **Table S2.** A summary of the functional classification of the 4004 hits based on the NCBI definition. **Table S3.** A summary of 66 samples of ten tissue types across different developmental stages sequenced by Illumina sequencer. **Table S4.** A summary of Spearman correlation coefficient between biological replicates for each of the ten tissue types. **Table S5.** A summary of the false positively aligned Sanger sequences to the genome based on our model. **Table S6.** A summary of the difference in genes annotated in Pvir_v3 and Pvir_v4 by PacBio transcripts after each comparison cycle.

## Abbreviations

PacBio: Pacific Biosciences
AS: alternatively spliced
FL: full length
Pvir_v3: switchgrass genome version 3
Pvir_v4: switchgrass genome version 4
CW: cell wall
ROI: reads of insert
IR: intron retention
A3SS: alternative 3′ splice site
A5SS: alternative 5′ splice site
ES: exon skipping
CPC: coding potential calculator
TF: transcription factor
GO: Gene Ontology
CCS: circular consensus sequences
