# Supplementary material for: Revealing the transcriptomic complexity of switchgrass by PacBio long-read sequencing
Source: Biotechnol Biofuels. 2018 Jun 20;11:170. doi: 10.1186/s13068-018-1167-z (PMC6009963; doi:10.1186/s13068-018-1167-z)
Supplement: Supplementary file 10 — Additional file 10: Figure S1. The distribution of the sequence lengths of ROI for each size-fractional library. Figure S2. The workflow of the Iso-seq Tofu pipeline. Figure S3. The workflow of the transcriptome analysis and assembly pipeline for PacBio transcripts. Figure S4. An illustrative example of fusion transcripts. Figure S5. A Venn diagram of our predicted 8850 fusion transcripts showing homology to proteins of the three related species. Figure S6. A Venn diagram of 1296 HQ FL transcripts showing homology to proteins of the three related species. Figure S7. Length distribution of FL and non-FL transcripts in each library. Figure S8. The base-pair mismatch rate of non-FL PacBio sequences in each size-fractional library. Figure S9. Validation of PacBio transcripts by Illumina-based RNA-seq reads. Figure S10. Illustrative examples of nine groups of PacBio transcripts. Figure S11. Validation of AS events by Illumina reads. Figure S12. Quantification of PacBio transcripts. Figure S13. Thresholds for selection of two parameters: the e value and the proportion of best-hit bit-score. Figure S14. GO-based pathway enrichment analysis of our predicted CW transcripts. Figure S15. Characterization of our predicted lncRNAs. Figure S16. 54 TF families identified from FL and non-FL transcripts. Figure S17. The distribution of the ratio between sequence lengths of 1174 transcripts in Pvir_v3 vs. Pvir_v4. Figure S18. Size-dependent cDNA libraries of Switchgrass. Figure S19. Number of sequencing pass for FL transcripts in each library. Figure S20. The probability (p value) that the number of errors (K) in a sequence of length L is larger than ($L(p + 3{\text{\% }})$L(p+3\%)). Figure S21. The distribution of base-pair mismatch rate of [file 13068_2018_1167_MOESM10_ESM.docx]

**Figure S1.** The distribution of the sequence lengths of ROI for each size-fractional library. (a) Gel. (b) SageELF. Each color indicates each of different library.

**Figure S2.** The workflow of the Iso-seq Tofu pipeline.

**Figure S3.** The workflow of the transcriptome analysis and assembly pipeline for PacBio transcripts.

**Figure S4.** An illustrative example of fusion transcripts. (a) Schematic of one group of transcripts having same boundary sites. (b) Improvement of current genome assembly and annotation by fusion transcripts. Note: (i) and (ii) indicate the schematic of the mapping of 6,878 and 1,972 inter and intra-chromosomal fusion transcripts at Pvir_v3, respectively; (iii) 6,878 and 1,972 inter and intra-chromosomal fusion transcripts are homologous to the proteins of three related species.

**Figure S5.** A Venn diagram of our predicted 8,850 fusion transcripts showing homology to proteins of the three related species. (a) 6,878 inter-chromosomal fusion transcripts. (b) 1,972 intra-chromosomal fusion transcripts.


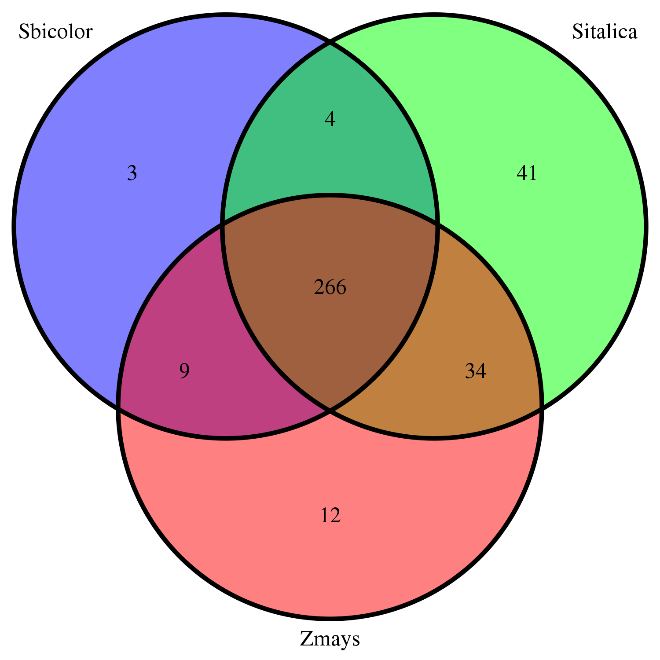


**Figure S6.** A Venn diagram of 1,296 HQ FL transcripts showing homology to proteins of the three related species.


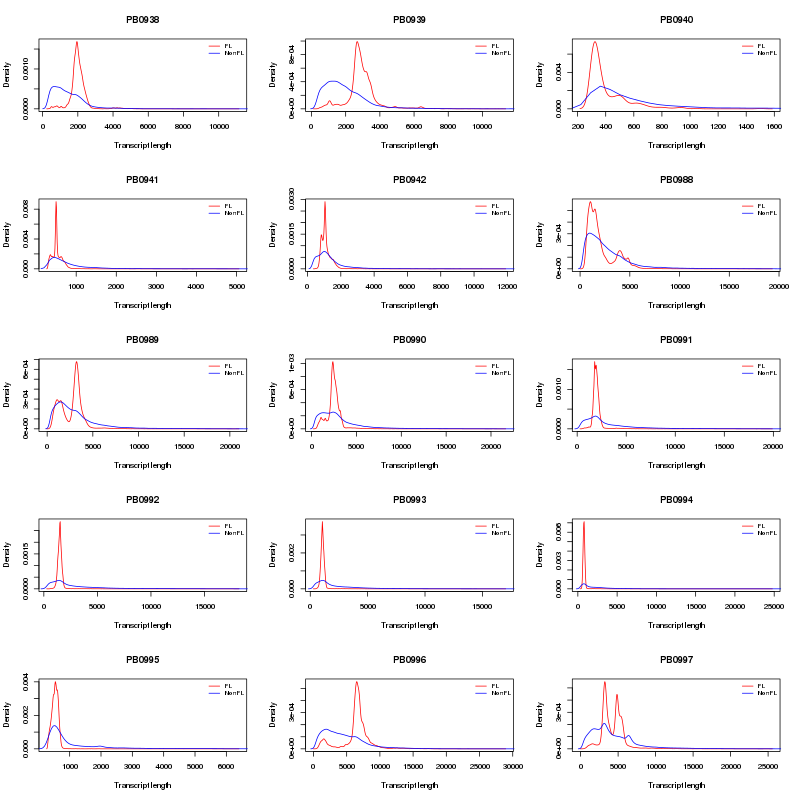


**Figure S7.** Length distribution of FL and non-FL transcripts in each library. For each sub panel, the red and blue lines indicate FL and non-FL transcripts, respectively.

**Figure S8.** The base-pair mismatch rate of non-FL PacBio sequences in each size-fractional library. (a) The mismatch rate of PacBio sequences in each library. The blue vertical line and the number denote the medium value of the error rate for each library. (b) The medium mismatch error rate as a function of medium length of sequence for each library.

**Figure S9.** Validation of PacBio transcripts by Illumina-based RNA-seq reads. (a) The percentage of splice junction of PacBio transcripts are supported by Illumina reads. The x-axis represents 66 samples across ten tissue types sequenced by Illumina sequencer and the y-axis represents the percentage of splice-junctions in PacBio transcripts with supporting evidence from Illumina reads in each sample. The color indicates different number of reads. (b) The percentage of splice junction of PacBio transcripts could be supported by different number of Illumina reads on average and in total, respectively.

**Figure S10.** Illustrative examples of nine groups of PacBio transcripts. (a) PacBio transcripts are missed from Pvir_v3. (b) PacBio transcripts each contain one Pvir_v3 gene. (c) PacBio transcript located inside an intron of a Pvir_v3 gene. (d) PacBio transcript has one intron that contains a Pvir_v3 gene. (e) PacBio transcript has same exon-intron structure with Pvir_v3 transcript. (f) PacBio transcript overlap with part of but not the whole exon of a Pvir_v3 gene on the opposite genomic strand. (g) PacBio transcript overlap with part of but not the whole exon of Pvir_v3 gene on the same strand. (h) PacBio transcript share at least one splice junction with Pvir_v3 gene, but differ at other splice sites. (i) PacBio transcript properly contained in the coding region of a Pvir_v3 gene. Note: the transcripts in Pvir_v3 and PacBio are marked with red and blue color, respectively.


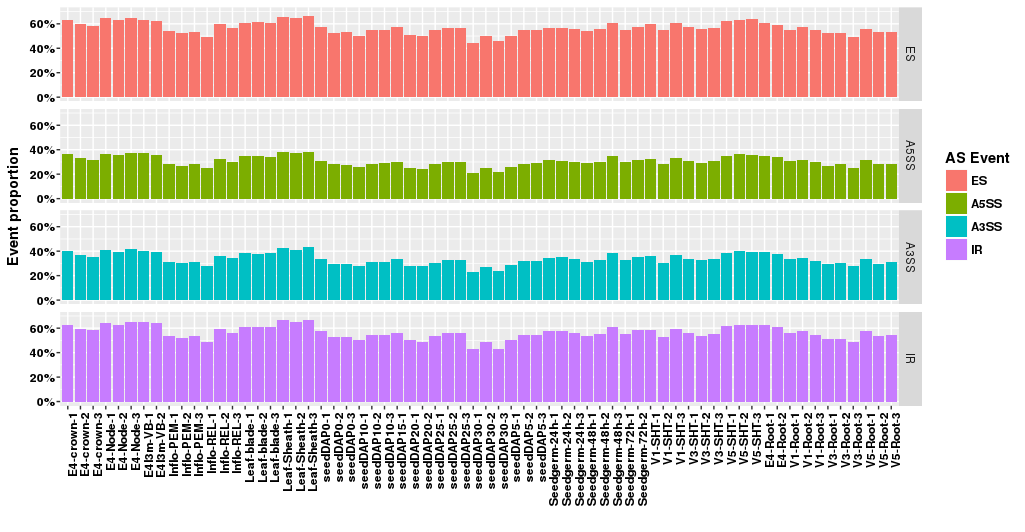


**Figure S11.** Validation of AS events by Illumina reads. The bar plot of the proportions of four types of AS events, supported by Illumina reads, across ten tissue types.

**Figure S12.** Quantification of PacBio transcripts. (a) Cutoff of expressions based on saturation analysis. (b) Principal component analysis of FPKM value of 52,880 transcripts across ten tissue types. The x-axis and y-axis indicates the first and second principal component, respectively. Each color represents a distinct tissue type.


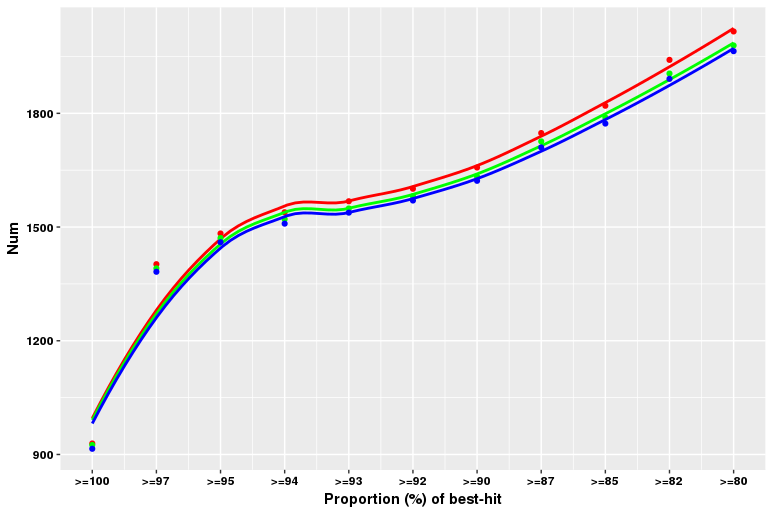


**Figure S13.** Thresholds for selection of two parameters: the e-value and the proportion of best-hit bit-score. The x-axis is the proportion of the bit-score of the best-hit regarded as CW-related transcripts. The y-axis is the number of transcripts for each corresponding proportion. The red, green and blue color lines indicate the e-value was less than 1E-10, 1E-20, 1E-30, respectively.

**Figure S14.** GO-based pathway enrichment analysis of our predicted CW transcripts. (a) Biological process. (b) Molecular function. (c) Cellular component. The p-value cutoff is 0.01.

**Figure S15.** Characterization of our predicted lncRNAs. (a) Venn diagram of discarded potential protein-coding transcripts from 13,021 candidate lncRNAs. (b) Venn diagram of our predicted 5,165 lncRNAs showing homology to lncRNAs in three related species. Note: no homology at Sitalica. (c) The number of exons per lncRNA vs. that per non-lncRNA. (d) Distributions of the FPKM values of lncRNA vs. non-lncRNA.

**a**


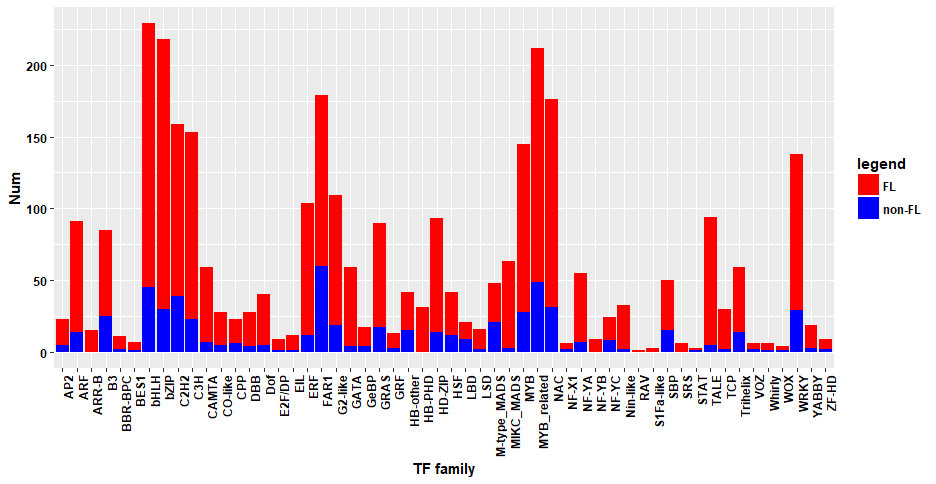


**Figure S16.** 54 TF families identified from FL and non-FL transcripts.


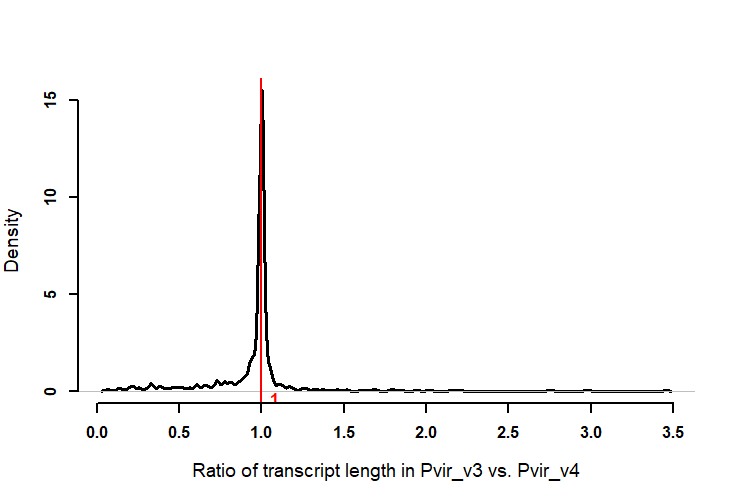


**Figure S17.** The distribution of the ratio between sequence lengths of 1,174 transcripts in Pvir_v3 vs. Pvir_v4.

**Figure S18.** Size-dependent cDNA libraries of Switchgrass. (a) Quantification of FL cDNA by Bioanalyzer, small size RNAs were observed. (b) Quantification of size-fraction libraries using gel by Bioanalyzer. Note: the color indicates different libraries. (c) Quantification of size-fraction libraries using SageELF by Bioanalyzer.


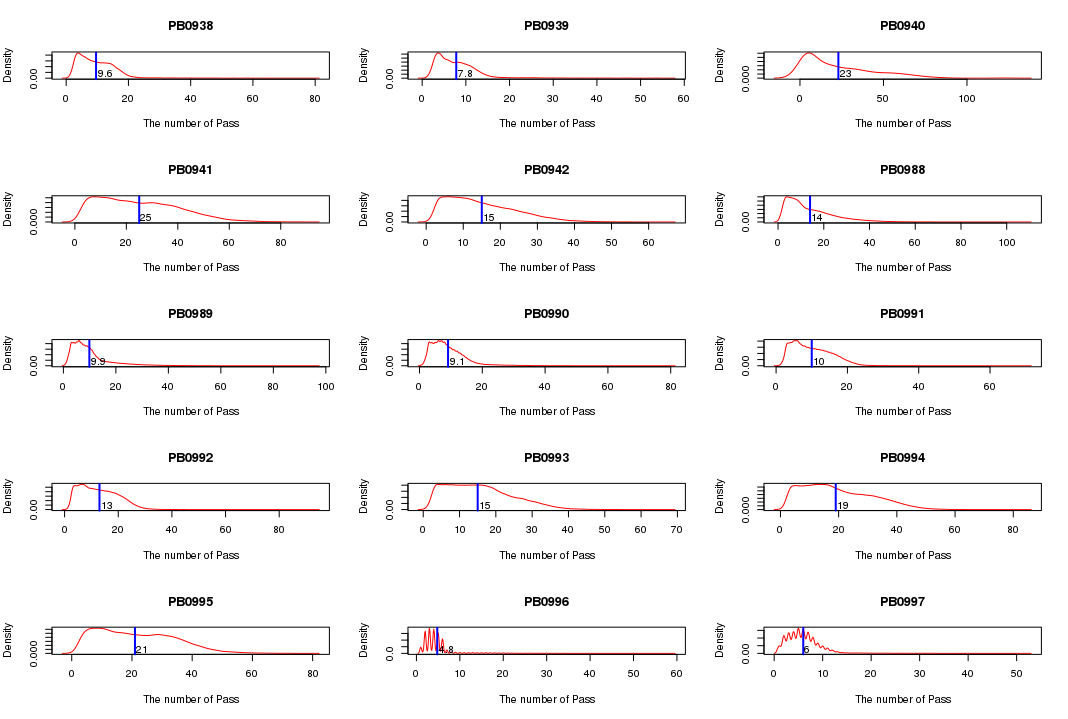


**Figure S19.** Number of sequencing pass for FL transcripts in each library. For each sub panel, the blue vertical line and the number denote the mean value of the pass number for each library.


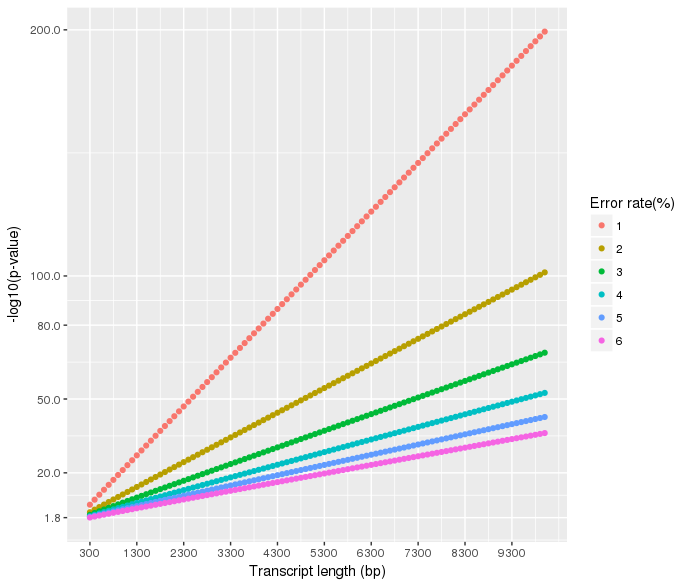


**Figure S20.** The probability (p-value) that the number of errors (*K*) in a sequence of length *L* is larger than ($L(p+3\%)$). The x-axis indicates the transcript length *L* range from 300 to 1kbp. The y-axis indicates p-value transformed by the function $-log10$. The color indicates different mismatch rate *p*.


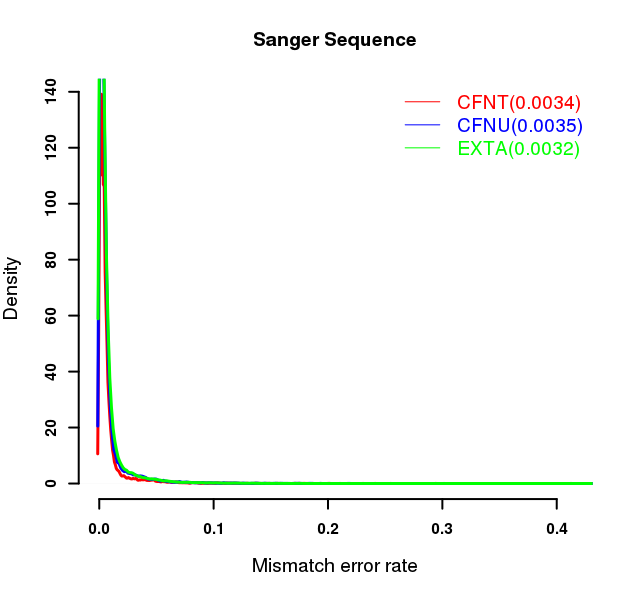


**Figure S21.** The distribution of base-pair mismatch rate of Sanger sequences. Three different libraries are marked with red, blue and green color, respectively. The number insider each pair of parentheses represents the medium of mismatch error rate for each library.


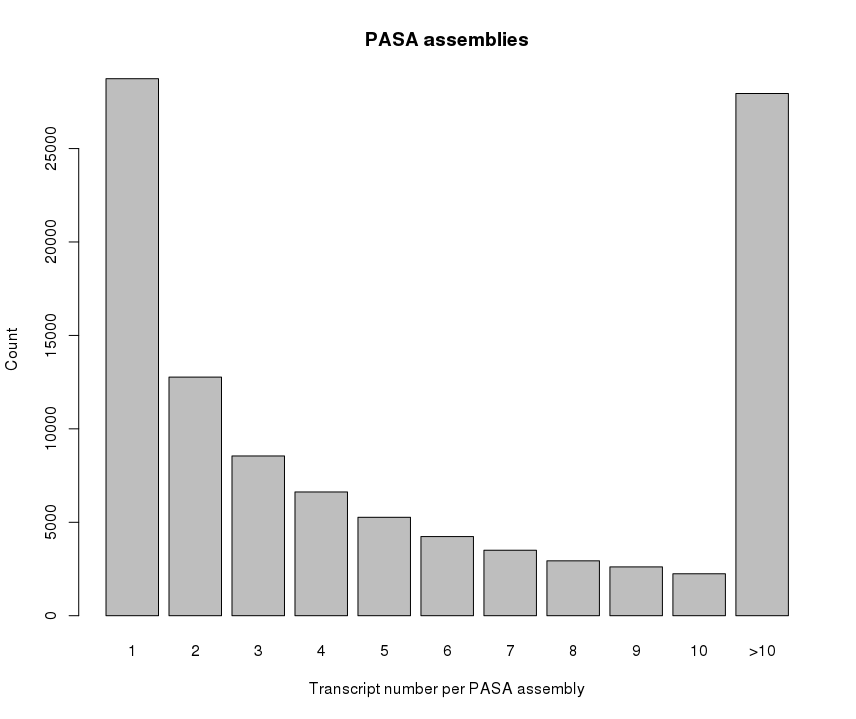


**Figure S22.** The bar plot of the number of transcripts collapsed by each PASA assembly.
