# Supplementary material for: Revealing the transcriptomic complexity of switchgrass by PacBio long-read sequencing
Source: Biotechnol Biofuels. 2018 Jun 20;11:170. doi: 10.1186/s13068-018-1167-z (PMC6009963; doi:10.1186/s13068-018-1167-z)
Supplement: Supplementary file 11 — Additional file 11: Table S1. A summary of six classifications based on the presence of 5′ primer, 3′ primer and polyA signal in each non-FL transcripts of different size-fractional library sets. Table S2. A summary of the functional classification of the 4004 hits based on the NCBI definition. Table S3. A summary of 66 samples of ten tissue types across different developmental stages sequenced by Illumina sequencer. Table S4. A summary of Spearman correlation coefficient between biological replicates for each of the ten tissue types. Table S5. A summary of the false positively aligned Sanger sequences to the genome based on our model. Table S6. A summary of the difference in genes annotated in Pvir_v3 and Pvir_v4 by PacBio transcripts after each comparison cycle. [file 13068_2018_1167_MOESM11_ESM.docx]

**Table S1.** A summary of six classifications based on the presence of 5’ primer, 3’ primer and polyA signal in each non-FL transcripts of different size-fractional library sets.

| **Library** | **#Non-FL reads** | **#5’ primer, poly A** | **#5’ primer, 3’ primer** | **#5’ primer** | **#3’ primer, poly A** | **#3’ primer** | **#None** |
| --- | --- | --- | --- | --- | --- | --- | --- |
| **PB0938** | 33,863 | 921 | 662 | 5,951 | 6,482 | 778 | 19,069 |
| **PB0939** | 41,604 | 720 | 252 | 6,012 | 7,444 | 888 | 26,288 |
| **PB0940** | 2,371 | 23 | 292 | 553 | 234 | 69 | 1,200 |
| **PB0941** | 11,806 | 728 | 1,741 | 2,862 | 2,730 | 208 | 3,537 |
| **PB0942** | 48,990 | 2,099 | 1,515 | 13,994 | 10,043 | 960 | 20,379 |
| **PB0988** | 156,094 | 3,701 | 4,836 | 31,325 | 39,315 | 3,431 | 73,486 |
| **PB0989** | 205,554 | 3,869 | 6,100 | 40,488 | 49,618 | 5,074 | 100,405 |
| **PB0990** | 205,387 | 4,612 | 6,544 | 37,467 | 54,415 | 5,507 | 96,842 |
| **PB0991** | 154,177 | 4,024 | 5,401 | 27,495 | 45,104 | 4,112 | 68,041 |
| **PB0992** | 140,062 | 3,947 | 4,769 | 24,035 | 40,936 | 3,908 | 62,467 |
| **PB0993** | 138,236 | 4,013 | 5,231 | 22,376 | 45,303 | 4,204 | 57,109 |
| **PB0994** | 116,597 | 3,769 | 5,690 | 17,808 | 39,577 | 3,747 | 46,006 |
| **PB0995** | 19,610 | 977 | 3,049 | 2,801 | 6,471 | 485 | 5,827 |
| **PB0996** | 249,333 | 1,091 | 12,210 | 63,684 | 29,104 | 5,124 | 138,120 |
| **PB0997** | 319,644 | 2,325 | 38,847 | 95,010 | 34,684 | 7,866 | 140,912 |
| **Total: 15** | 1,843,328 | 36,819 | 97,139 | 391,861 | 411,460 | 46,361 | 859,688 |
| **Proportion (%)** |  | 2 | 5 | 21 | 22 | 3 | 47 |

Each column indicates each of such transcript have this signal. Specifically, “#5’ primer, poly A” indicates such transcript contain both 5’ primer and poly A signal; and “None” indicates such transcript contain none of three signals.

**Table S2.** A summary of the functional classification of the 4,004 hits based on the NCBI definition.

| **Potential function/cellular organelle** | **# of hits** |
| --- | --- |
| retrotransposons | 933 |
| putative polyprotein | 394 |
| hypothetical protein | 281 |
| gag-pol like proteins | 198 |
| uncharacterized/unknown function | 158 |
| reverse transcriptases | 91 |
| transposases | 67 |
| chloroplast | 61 |
| helicases | 36 |
| zinc finger proteins | 28 |
| mitochondria | 20 |
| transporter proteins | 18 |
| disease resistance proteins | 14 |
| cytochrome p450 | 9 |
| Rnases | 9 |
| transcription factors | 9 |
| oxidoreductases | 7 |
| ribosomal proteins | 6 |
| serine carboxypeptidases | 3 |
| proline-rich proteins | 2 |
| Heat-shock proteins | 2 |
| secretory proteins | 2 |
| receptor-like protein kinases | 1 |
| tetratricopeptides | 1 |
| others | 1,654 |

**Table S3**. A summary of 66 samples of ten tissue types across different developmental stages sequenced by Illumina sequencer.

| **Tissue type** | **Samples design** | **# Sample** |
| --- | --- | --- |
| **Whole seed** | 24h, 48h, 72h | 3, 3, 2 |
| **Whole shoot** | V1, V3, V5 | 3, 3, 3 |
| **Whole root** | V1, V3, V5, E4 | 3, 3, 3, 2 |
| **Leaf blade** | Leaf blade (from E4 tiller) | 3 |
| **Leaf sheath** | Leaf sheath (from E4 tiller) | 3 |
| **Nodes** | E4 | 3 |
| **Vascular bundle** | E4i3m-VB | 2 |
| **Crown** | E4 stage | 3 |
| **Inflorescence** | REL, PEM | 3, 3 |
| **Flower & seed** | DAP0, DAP5, DAP10, DAP15, DAP20, DAP25, DAP30 | 3, 3, 3, 1, 2, 3, 3 |

The tissue type “whole seed” indicates the seed across different developmental stages (24h, 48h and 72h) of seed germination. The tissue type “Flower & seed” indicates seed and flower across seven development stages of seed growth, which is abbreviated as seedDAP. The tissue type for whole shoot, vascular bundle was abbreviated as SHT and VB, respectively.

**Table S4.** A summary of Spearman correlation coefficient between biological replicates for each of the ten tissue-type.

| **Tissue-type group** | **Range for correlation** |
| --- | --- |
| **Seedgerm-24h** | 0.967-0.971 |
| **Seedgerm-48h** | 0.942-0.971 |
| **Seedgerm-72h** | 0.971-0.971 |
| **seedDAP0** | 0.973-0.975 |
| **seedDAP5** | 0.973-0.974 |
| **seedDAP10** | 0.97-0.973 |
| **seedDAP20** | 0.966-0.966 |
| **seedDAP25** | 0.965-0.969 |
| **seedDAP30** | 0.87-0.963 |
| **V1-Root** | 0.966-0.969 |
| **V3-Root** | 0.964-0.969 |
| **V5-Root** | 0.962-0.965 |
| **E4-Root** | 0.966-0.966 |
| **LSH** | 0.96-0.963 |
| **Leaf** | 0.965-0.969 |
| **VB** | 0.971-0.971 |
| **crown** | 0.965-0.969 |
| **Inflo-REL** | 0.961-0.969 |
| **Inflo-PEM** | 0.96-0.968 |
| **Node** | 0.967-0.969 |
| **V1-SHT** | 0.966-0.971 |
| **V3-SHT** | 0.963-0.966 |
| **V5-SHT** | 0.975-0.976 |

**Table S5.** A summary of the false positively aligned Sanger sequences to the genome based on our model.

| **Type** | **Chromosome** | | | | | | | | | |
| --- | --- | --- | --- | --- | --- | --- | --- | --- | --- | --- |
|  | **Chr01** | **Chr02** | **Chr03** | **Chr04** | **Chr05** | **Chr06** | **Chr07** | **Chr08** | **Chr09** |  |
| **#mapped Sanger sequence** | 143,770 | 143,861 | 150,042 | 153,689 | 139,698 | 153,787 | 151,885 | 157,351 | 136,521 |  |
| **unmapped proportion (%)** | 91.4 | 90.5 | 90.5 | 93.7 | 89.3 | 93.3 | 93.2 | 93.4 | 88.3 |  |
| **False positive (%)** | 14 | 15 | 17 | 16 | 20 | 17 | 18 | 18 | 21 |  |

**Table S6.** A summary of the difference in genes annotated in Pvir_v3 and Pvir_v4 by PacBio transcripts after each comparison cycle.

|  | | **Cycle** | | | |
| --- | --- | --- | --- | --- | --- |
|  |  | First | Second | Third | Four |
| **Pvir_v3** | **#Loci in genome** | 16,635 | 16,640 | 16,640 | 16,640 |
|  | **#PacBio transcripts** | 19,613 | 19,938 | 20,018 | 20,041 |
| **Pvir_v4** | **#loci in genome** | 16,539 | 16,544 | 16,544 | 16,544 |
|  | **#PacBio transcripts** | 19,507 | 19,822 | 19,903 | 19,926 |
